# Classification of Cardiotocography Based on the Apriori Algorithm and Multi-Model Ensemble Classifier

**DOI:** 10.3389/fcell.2022.888859

**Published:** 2022-05-11

**Authors:** Meng Chen, Zhixiang Yin

**Affiliations:** School of Mathematics, Physics and Statistics, Shanghai University of Engineering Science, Shanghai, China

**Keywords:** apriori, multi-model integration, CTG (cardiotocography), classification, AdaBoost

## Abstract

Cardiotocography (CTG) recorded fetal heart rate and its temporal relationship with uterine contractions. CTG intelligent classification plays an important role in evaluating fetal health and protecting fetal normal growth and development throughout pregnancy. At the feature selection level, this study uses the Apriori algorithm to search frequent item sets for feature extraction. At the level of the classification model, the combination model of AdaBoost and random forest with the highest classification accuracy is finally selected by comparing various models. The suspicious class data in the CTG data set affect the overall classification accuracy. The number of suspicious class data is predicted by the multi-model ensemble method. Finally, the data set is fused from three classifications to two classifications. The classification accuracy is 0.976, and the AUC is 0.98, which significantly improves the classification effect. In conclusion, the method used in this study has high accuracy in model classification, which is helpful to improve the accuracy of fetal abnormality detection.

## 1 Introduction

A fetal electrocardiogram is a map of the biological current generated by the instantaneous change of fetal heart activity. By observing the cardiac electrical activity, the fetal heart rhythm can be accurately detected, and fetal arrhythmia can be clearly classified. In order to timely detect whether the intrauterine environment of the fetus has changed and determine which fetus may be hypoxic, so as to remind clinicians to carry out intervention treatment, the incidence of a newborn should be reduced, and the prenatal healthcare level should be improved, to ensure the safety of the mother and child. Cardiotocography is generally divided into three types: normal, suspect, and pathologic.

In (Ocak, 2013; Ocak and Ertunc, 2013; Chen et al., 2021), predicting the fetal status based on CTG data is regarded as a binary classification problem. In (Ocak and Ertunc, 2013), an adaptive neuro-fuzzy inference system (ANFIS) was used to predict the fetal state based on electrocardiogram records. In (Ocak, 2013), a hybrid system based on the combination of the support vector machine (SVM) and the genetic algorithm (GA) is proposed to make medical decisions for fetal health assessment. Chen et al. (2021) proposed an intelligent classification of the antepartum cardiotocography model based on deep forest, which solved the problem of the high misjudgment rate of normal and suspicious classification.

In (Yılmaz and Kılıkçıer, 2013; Sindhu et al., 2015; Yılmaz, 2016), the determination of the fetal status based on CTG data is also modeled as a three classification problem. Yılmaz (2016) used three artificial neural network models, namely, the multi-layer perceptron neural network (MLPNN), probabilistic neural network (PNN), and generalized regression neural network (GRNN), to compare the evaluation of the fetal state and concluded that the PNN network model had the best overall classification effect. In Sindhu et al. (2015), the author proposed a new clinical decision support system based on the improved adaptive genetic algorithm (IAGA) and the Extreme Learning Machine (ELM) algorithm, and the final classification accuracy of the model reached 94%. In Yılmaz and Kılıkçıer (2013), based on the least squares support vector machine (LS-SVM), the particle swarm optimization (PSO) and the binary decision tree (BDT) were combined to optimize the parameters so as to determine the fetal status of the electrocardiogram.

In Zeng et al. (2021), the author introduces a classifier based on the time–frequency (TF) feature and integrated a cost-sensitive support vector machine (ECSVM). The non-stationarity of CTG and the imbalance of the data set are solved, and it obtains more effective results, with a sensitivity of 85.2%, specificity of 66.1%, and quality index of 75.0%. In Improta et al. (2019), the author used the self-developed CTG automatic analysis software to extract feature data from CTG signals and predict childbirth through different algorithms: J48, AdaBoosting, random forests, and gradient boosting tree. The results of RF classification reached the highest with accuracy = 87.6% and AUCROC = 93.0%. Ricciardi et al. (2020) extracted 17 features from existing CTG signals using customized software and classified them using a machine learning algorithm: J48, random forest (RF), and decision tree AdA-Boosting (AdA-B), in which RF and AdA-B obtained better classification results with AUCROC greater than 94.9%. In Amin et al., (2021), the author proposed an interval neutrophil rough neural network framework based on the backpropagation algorithm and compared it with other algorithms: neural network, decision tree, K-nearest neighbor, and rough neural network; this framework is a feasible and efficient classifier. In Jeżewski et al., (2014), the author mainly carried out different feature selection methods through principal component analysis, recipient operating characteristics, and the International Federation of Obstetrics and Gynecology guidelines and verified the impact of the application on the quality of fetal state assessment by using the benchmark SisPorto data set and Lagrange support vector machine. In Impey et al. (2003), compare the effect on neonatal outcome of admission cardiotocography *versus* intermittent auscultation of the fetal heart rate. Explain that routine use of ecg for 20 min at admission does not improve neonatal outcomes. In de l’Aulnoit et al. (2018), the authors compared 11 morphological FHR analyses (baseline calculations, and detection of FHR deceleration and acceleration) generated by the automatic analysis method (AAM) with expert consensus. Conclusion: The AAM developed by Lu and Wei provided better results in baseline calculation than other AAms. In Grivell et al. (2012), assess the effectiveness of antenatal CTG (both traditional and computerised assessments) in improving outcomes for mothers and babies during and after pregnancy. In Alfirevic et al. (2013), cardiotocography aim is to identify babies who may be short of oxygen (hypoxic), so additional assessments of fetal well-being may be used, or the baby delivered by caesarean section or instrumental vaginal birth. In Devane et al. (2012), the authors will use randomized and semi-randomized trials to compare admission CTG with intermittent fetal heart auscultation at 37 to 42 weeks of gestation, suggesting a lower risk of fetal hypoxia and delivery complications. In Molla et al. (2021), the random forest (RF) algorithm is used to classify CTG data. The results show that the RF-based classifier can identify normal, suspicious and pathological states from the properties of CTG data with an accuracy of 94.8%. In Gatellier et al. (2021), fetal well-being during labor is usually assessed by visual analysis of a fetal heart rate (FHR) tracing. The authors’ main aim was to evaluate the ability of automated heart rate variability (HRV) analysis methods. In Silwattananusarn et al. (2020), the feature selection method based on integration is applied to select the feature set which may be support vector, and the SVM integration algorithm is constructed using the selected features. The proposed method evaluates experiments with the Cardiotocography dataset. In Subasi et al. (2020), this paper focuses on Bagging integrated machine learning algorithms for classifying fetal heart rate signals as normal or abnormal. The experimental results show that Bagging with Random Forest achieves better results with an accuracy of 99.02%.

In this study, feature extraction is carried out by the Apriori algorithm. By predicting suspicious data, the crossover problem between suspicious data sets and health and pathology data sets is solved, and the classification accuracy is improved.

The literature (Yılmaz, 2016; Chen et al., 2021) clearly points out that there are crossover problems between suspicious data and normal and pathological data, which affect the accuracy of classification. In this study, feature extraction is carried out by the Apriori algorithm, and suspicious data are predicted and classified by two models. The crossover problem between suspicious data sets and health and pathology data sets is solved, and the classification accuracy is improved. The two models are complementary to each other. On the one hand, the prediction accuracy of model 2 is high, and on the other hand, model 1 is correct.

## 2 Materials and Methods

### 2.1 Cardiotocography dataset

CTG datasets are derived from publicly available datasets in the UCI Machine learning library (http://archive.ics.uci.edu/ml/datasets/Cardiotocography). The dataset includes measurements of fetal heart rate (FHR) and uterine contraction (UC) characteristics on fetal heart charts classified by specialist obstetricians. There are 2, 126 sample real numbers and 23 attribute descriptions in the dataset. The last column is the category label, where 1 is healthy, 2 suspicious, and 3 pathological. Table 1 shows the list of attributes.

**TABLE 1:** Attributes of the CTG dataset.

Number	Attribute	Definition
1	LB	Baseline value (SisPorto)
2	AC	Acceleration (SisPorto)
3	FM	Fetal movement (SisPorto)
4	UC	Uterine contraction (SisPorto)
5	ASTV	Percentage of time with abnormal short-term variability (SisPorto)
6	mSTV	Mean value of short-term variability (SisPorto)
7	ALTV	Percentage of time with abnormal long-term variability (SisPorto)
8	mLTV	Mean value of long-term variability (SisPorto)
9	DL	Light decelerations
10	DS	Severe decelerations
11	DP	Prolonged decelerations
12	DR	Repetitive decelerations
13	Width	Histogram width
14	Min	Low frequency of the histogram
15	Max	High frequency of the histogram
16	Nmax	Number of histogram peaks
17	Nzeros	Number of histogram zeros
18	Mode	Histogram mode
19	Mean	Histogram mean
20	Median	Histogram median
21	Variance	Histogram variance
22	Tendency	Histogram tendency: 1 = left asymmetric; 0 = symmetric; 1 = right asymmetric
23	NSP	Normal = 1; Suspect = 2; Pathologic = 3

### 2.2 Feature Selection

Biomedical expression data possess the characteristics of unbalanced distribution, high dimensionality, small sample, and high noise. Direct classification is not only time-consuming but also has low classification accuracy; therefore, feature selection is needed for dimensionality reduction and redundancy processing. In this study, the filtering method is adopted as the method of feature selection. The chi-squared test is performed on 1–22 attributes, DR P (k-w) = 1, so DR is removed. There are 21 attributes used in this study, and the 22nd is the category label.

#### 2.2.1 Apriori Algorithm

The association analysis is an unsupervised learning algorithm for finding relationships in large-scale datasets. This relationship can take two forms: frequent item sets or association rules. Frequent itemsets are collections of items that occur together frequently, and association rules imply that there may be a strong relationship between two items.

The evaluation criteria of frequent itemsets include support, confidence, and promotion (support is used in this study). Support refers to the proportion of the number of occurrences of several related data in the data set to the total data set. For data *X* and *Y* of two correlations, the corresponding support degree is

$$\text{Support}\left(\text{X},\text{Y}\right)=\text{P}\left(\text{X},\text{Y}\right)=\frac{\text{number}\left(\text{XY}\right)}{\text{num}\left(\text{AllSamples}\right)}.$$ (1)

In this study, the Apriori algorithm is used for association analysis. The principle of the Apriori algorithm is if an itemset is frequent, then all its subsets must also be frequent. The specific steps in this study are shown in Algorithm 1.

Algorithm 1 Input: CTG feature attributes

Output: feature attribute column

Step 1 encode each column of attributes

Step 2 mine frequent itemsets

Step 3 extract feature attribute columns according to Step 2

Step 4 stop

In this study, the association analysis is carried out on the basis of mutual information. By mining frequent itemsets, we carried out set operations on the attribute columns of healthy frequent itemsets, suspicious frequent itemsets, and pathological frequent itemsets and finally screened out 13 feature attributes closely related to category labels.

### 2.3 Model Construction

The k-nearest neighbor (KNN) algorithm learns according to class comparison. Working principle: given a training data set with a known label category, the new data should be entered without a label; K instances closest to the new data are found in the training data set. If most of the K instances belong to a certain category, then the new data will fall into this category. It can be simply understood as follows: the *k* points closest to *X* vote to determine which category *X* belongs to. The k-nearest neighbor algorithm is different from most machine learning algorithms; it does not need iterative optimization parameters but only needs to select appropriate *K* values to specify the samples to be classified. In the process of training the k-nearest neighbor algorithm, the definition of distance, the selection of K value, and classification decision rules are very important.

The Bayesian classification algorithm is a probability classification method in statistics. Classification principle: Bayesian formula is used to calculate the posterior probability according to the prior probability of a feature, and then, the class with the maximum posterior probability is selected as the class to which the feature belongs. GaussianNB (GNB) is a naive Bayes whose priors are Gaussian distributions, assuming that the data of each label obey a simple normal distribution. Gaussian naive Bayes performs well on small-scale data and can handle multi-classification tasks.

$$P\left(X_{j}=x_{j}|Y=C_{k}\right)=\frac{1}{\sqrt{2\pi \sigma_{k}^{2}}}\exp\left(-\frac{{\left(x_{j}-u_{k}\right)}^{2}}{2\sigma_{k}^{2}}\right),$$ (2)

where $C_{k}$ is the Y category. $u_{k}$ and $\sigma_{k}^{2}$ are the estimated values of the training set.

Logistic regression function is a differentiable convex function of any order, which has good solving properties. Logistic regression can handle nonlinear classification tasks without assuming data distribution. In the machine learning algorithms, the stochastic gradient descent (SGD) is to find the minimum value of the function along the opposite direction of the gradient vector (i.e., the fastest gradient reduction). SGD does not use all the sample data but only selects a sample J to calculate the gradient. Its updated formula is as follows:

$$\theta_{i}=\theta_{i}-\alpha \left(h_{\theta }\left(x_{0}^{\left(j\right)},x_{1}^{\left(j\right)}\cdots x_{n}^{\left(j\right)}\right)-y_{j}\right)x_{i}^{\left(j\right)}.$$ (3)

SGD uses only one sample iteration at a time, so the training speed is very fast. It performs well in the process of nonconvex function optimization. Because of the randomness of its descending direction, it can well bypass the local optimal solution and approach the global optimal solution.

Ensemble learning is divided into bagging and boosting. Boosting is an iterative process, which adaptively changes the distribution of training samples to make the weak classifiers focus on the samples that are difficult to classify. It does this by assigning a weight to each training sample and automatically adjusting the weight at the end of each training round. AdaBoost is an acronym for Adaptive Boosting. In the learning process of AdaBoost, the weight of each classifier is fully considered, which can better predict the class markers of new samples and improve the accuracy and stability of the ensemble classifier. The number of AdaBoost iterations can be determined by cross-validation.

#### 2.3.1 Model One

Model 1 includes five machine learning algorithms: k-nearest neighbor, GaussianNB, SGD, AdaBoost, and AdaBoost combined with random forest (Ada-RF); these five algorithms randomly divide the training set and test set (training set 0.75 and test set 0.25), in which the maximum number of model iterations is 10,000.

**AdaBoost:** n_ESTIMators = 100, learning_rate = 0.5, algorithm = “samme. R”, and random state = 25.

**Ada-RF:** random forest classifier (n_ESTIMators = 1, 000), learning_rate = 0.5, algorithm = “SAMME”, and n_ESTIMators = 500.

For (Yılmaz, 2016; Chen et al., 2021), it is proposed that there is a crossover problem between normal classes and doubtful classes, which affects the accuracy of classification results. In this study, health and suspicious, health and pathology, and suspicious and health dichotomies are established. According to model 1, the classification model of this study is determined. At the same time, whether there are crossover problems between suspicious, healthy, and pathology is verified.

#### 2.3.2 Model Two

According to model 1, the influence of suspicious class data on the classification model is found. This study proposes a secondary learning model based on a multi-model ensemble feedback machine for the health and pathological classification prediction of suspicious data. Model 2 mainly uses the k-nearest neighbor, GaussianNB, SGD, and AdaBoost models to perform the integrated operation and determine the final prediction results.

### 2.4 Evaluation Indicators

To evaluate the performance of the proposed method, the confusion matrix was used to evaluate each performance (Table 2). The samples were divided into true positive (TP), true negative (TN), false positive (FP), and false negative (FN), where TP + FN + FP + TN = a total number of samples. The performance indicators used in this article are as follows:

$$Accuracy=\frac{TP}{TP+FP},$$ (4)

$$Sensitivity=\frac{TP}{TP+FN},$$ (5)

$$F1=\frac{2\times Accuracy\times Sensitivity}{Accuracy+Sensitivity}.$$ (6)

**TABLE 2:** Confusion matrix of classification results.

Real results	Prediction results	
	Normal	Abnormal
Normal	TP	TN
Abnormal	FP	FN

## 3 Results and Discussion

### 3.1 Feature Selection Results and Discussion

**FIGURE 1:**
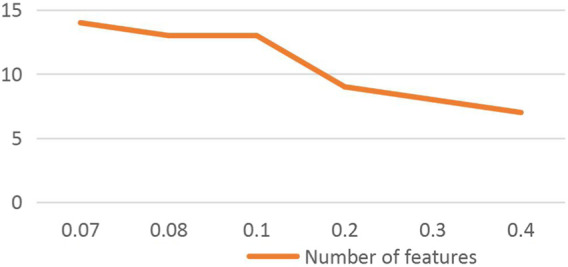
Number of feature extraction corresponding to minsupport.

At the feature selection level, all DS data of text data are 0. In order to ensure the rationality of data elimination, mutual information is used to eliminate attribute and label redundancy. Table 3 lists the redundant values for each attribute and table. On the basis of mutual information, we used the Apriori algorithm to extract frequent attribute columns by searching frequent itemsets. Table 4 lists the columns of attributes corresponding to frequent itemsets related to health, suspicion, and pathology. For medical pathological data, we should be very careful in attribute selection. Therefore, minsupport = 0.1 in the frequent itemset (Figure 1).

**TABLE 3:** Mutual information values of attributes and labels.

LB	AC	FM	UC	DL	DS	DP
0.1408	0.1398	0.0654	0.0650	0.0448	0.0058	0.0890

**ASTV**	**MSTV**	**ALTV**	**MLTV**	**Width**	**Min**	**Max**
0.2185	0.2353	0.2213	0.1658	0.2057	0.1846	0.1027

**Nmax**	**Nzeros**	**Mode**	**Mean**	**Median**	**Variance**	**Tendency**
0.0244	0.0069	0.1884	0.2038	0.1741	0.1969	0.0216

**TABLE 4:** Frequent itemset attribute columns of different categories.

Health	3	4	6	9	19	14	5	2	—	—	—	—	—
Suspicion	2	4	6	19	14	5	2	8	18	—	—	—	—
Pathology	3	4	6	9	19	14	5	2	8	18	10	12	1

Figure 1 shows that with the gradual increase of minsupport, the number of feature extraction gradually decreases. When minsupport is 0.08 and 0.1, the number of feature extraction is equal. By comparing the three features of healthy, suspicious, and pathological, when minsupport = 0.1, pathological features include healthy and suspicious features. Therefore, minsupport = 0.1 was determined. Table 4 lists the extracted feature attribute columns.

According to the mutual information value in Table 3, the greater the mutual information value, the stronger is the correlation between the two. With the threshold value of 0.01, DS (times of serious deceleration per second) and Nzeros (number of zeros in the histogram) with weak correlation were eliminated. The attribute data of DS were all 0, so it had no influence on the identification of labels and could be eliminated.

In Table 4 we extracted the frequent attribute columns related to health, suspicious, and pathology, respectively. It can be seen that the frequent itemset attribute column of pathology contains the health and suspicious attribute columns. Therefore, we performed a set calculation on them and finally extracted 13 feature attributes for the model.

They are as follows: LB, AC, FM, UC, DL, DP, MSTV, ALTV, MLTV, Min, Nmax, variance, tendency, and NSP.

### 3.2 Results and Discussion of Model One

We pairwise combined healthy, suspicious, and pathological data to compare the accuracy of dichotomies. In order to obtain the optimal classification accuracy, we selected KNN, GNB, SGD, AdaBoost, and AdaBoost combined with random forest (Ada-RF) and other machine learning models and selected Ada-RF with the best classification effect as the classification model in this study. The results are shown in Table 5.

**TABLE 5:** Classification accuracy table of data sets on different models.

	Raw data	Feature extraction	Raw data	Feature extraction	Raw data	Feature extraction
Combine	13	13	12	12	23	23
KNN	98.03%	93.89%	92.01%	90.16%	94.07%	93.22%
GNB	94.32%	93.68%	85.45%	87.09%	88.98%	84.74%
SGD	96.94%	93.01%	90.78%	91.39%	91.53%	91.53%
AdaBoost	98.25%	98.47%	93.85%	93.62%	97.45%	94.91%
Ada-RF	98.69%	98.47%	96.31%	94.88%	97.45%	96.61%

The GTC data category label includes one healthy, two suspicious, and three pathological conditions. Here, 13 represents the combination of healthy and pathological conditions, 12 represents the combination of healthy and suspicious conditions, and 23 represents the combination of suspicious and pathological conditions.

Table 5 shows the classification accuracy of the original attribute data set and the data set after feature extraction on each model, respectively, and the comparison between the two shows that the classification accuracy is generally the same. The classification accuracy of attributes after feature extraction is not significantly improved in Model 1, but the overall running time is greatly shortened, especially the Ada-RF is shortened from 68.46 to 36.78 s, which is about 31.68 s. We reduced the attribute dimension, reduced redundancy, and saved running time while keeping the classification accuracy unchanged. It shows that the feature extraction method in this study is feasible and effective.

**FIGURE 2:**
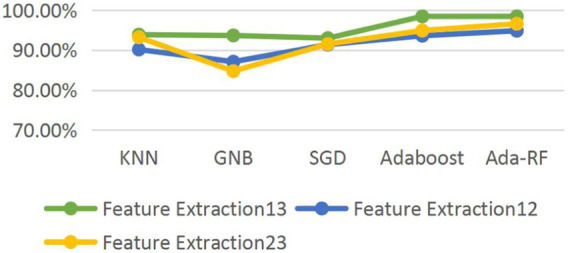
Line chart of the classification accuracy of the data set on different models.

Figure 2 shows the line chart of health and pathology, health and suspicious, suspicious and pathological combination classification accuracy after feature extraction. It is found that the classification accuracy of health and pathology combination is significantly higher than that of health and suspicious and suspicious and pathological combination. Verifying the statement of literature (Yılmaz, 2016; Chen et al., 2021), suspicious class data and normal, there is a cross between pathological data problems, affecting the accuracy of classification. We should further classify suspicious data to improve the classification accuracy of the whole data set. Ada-RF shows high classification results in both pairwise combinations, and the classification accuracy did not decrease after feature extraction and relatively improved operation time. Therefore, Ada-RF is determined to be the classification model of this study.

**FIGURE 3:**
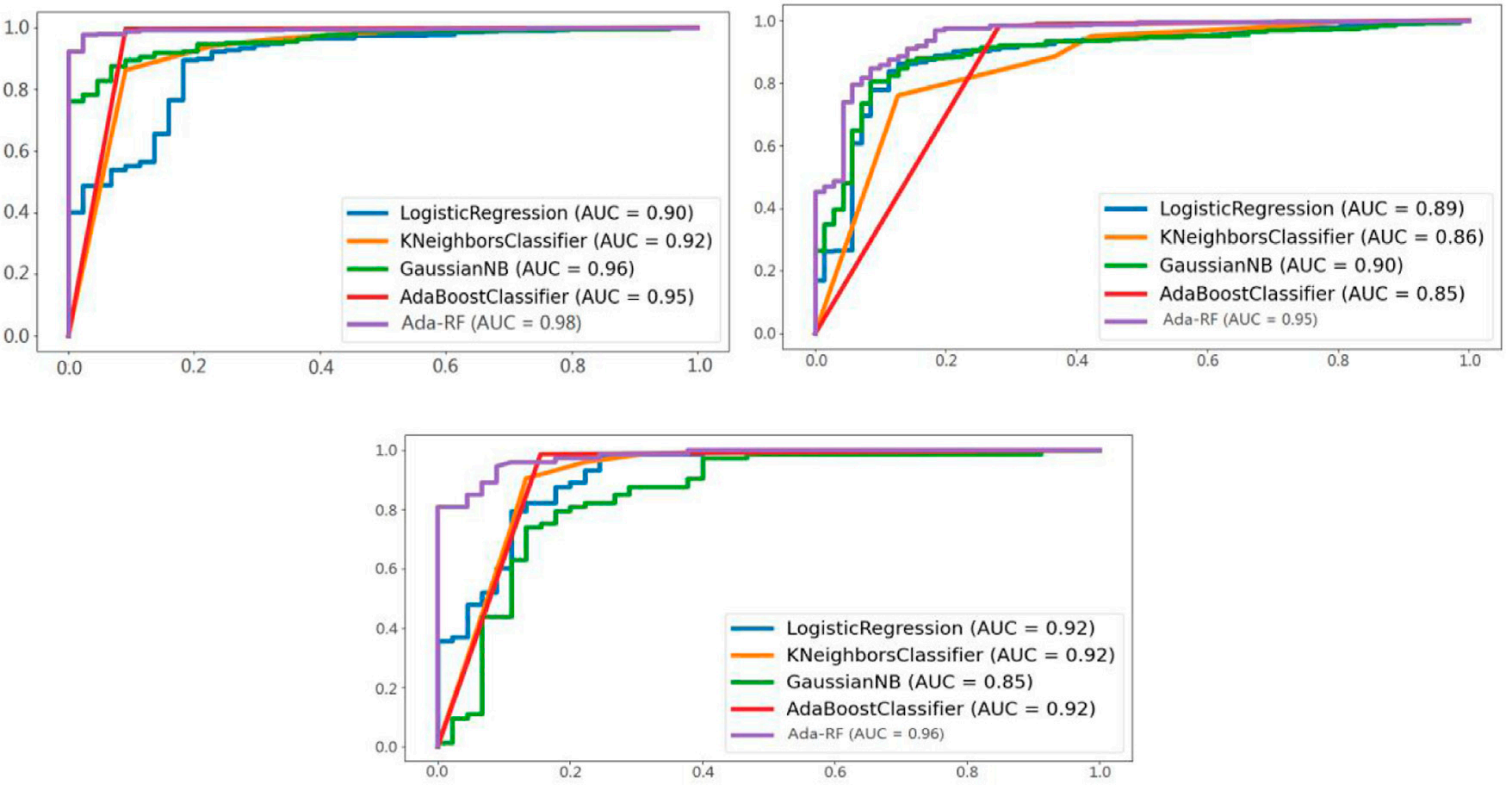
ROC curve areas of different models in the three groups of classification.

The confusion matrix is used to evaluate our proposed models and the ROC curve area diagram of different models in pair classification (Figure 3).

Figure 3 shows a comparison of the ROC curve area products of Ada-RF and other models in healthy versus pathological, healthy versus suspicious, and suspicious versus pathological. It can be seen from the figure that the ROC curves of Ada-RF models in different combinations are higher than those of other models. The AUC area of Ada-RF is also the largest among all models. The results show that the prediction performance of the Ada-RF model is better than other machine learning models.

### 3.3 Results and Discussion of Model Two

In the comparison of classification results of different models in Figure 2, we chose the Ada-RF model as the classification model for this study. The other four models have different classification accuracies in each combination. Therefore, when predicting suspicious data, we chose KNN, GNB, SGD, and AdaBoost models for integration calculation. Different machine learning algorithms are integrated to classify and predict suspicious data, and multiple highly differentiated classification results are obtained through training. The classification results of each model are integrated to obtain the predicted classification results of the final suspicious samples (Table 6).

**TABLE 6:** Classification accuracy of prediction results of different models.

	KNN (%)	GNB (%)	SGD (%)	AdaBoost (%)
Single model	78.98	59.67	55.59	90.51
Model 2	87.80	76.61	72.20	91.75

**FIGURE 4:**
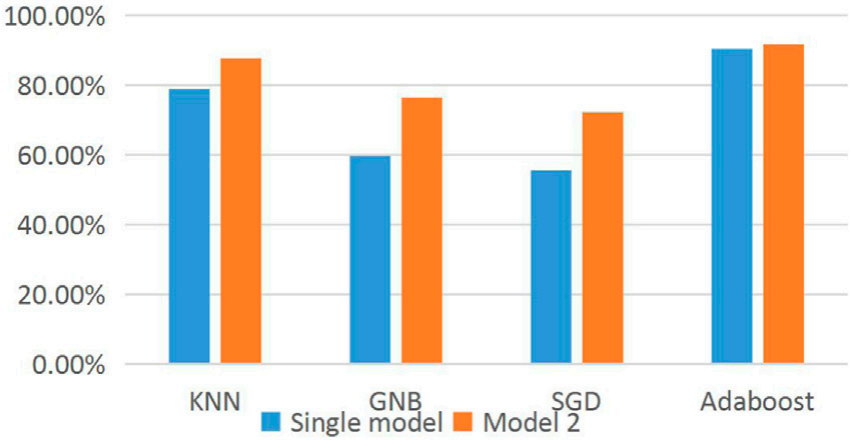
Histogram of the classification accuracy of prediction results of different models.

It can be seen from Figure 4 that the classification accuracy of all models has been significantly improved through the classification and prediction of suspicious data in model 2. KNN, GNB, and SGD increase by 14.12% on average and AdaBoost prediction by 1.24%, which proves that the prediction result of multiple models is better than that of a single model.

Through model 2, we have obtained the prediction results of suspicious data. The Ada-RF model is used to verify the prediction results, and all suspicious data are regarded as health or pathological data for comparison with the prediction results of model 2 (Table 7).

**TABLE 7:** Data set classification accuracy table.

	Suspicious as normal (%)	Suspicious as pathology (%)	Model 2 (%)
Ada-RF	96.80	93.98	97.55

**FIGURE 5:**
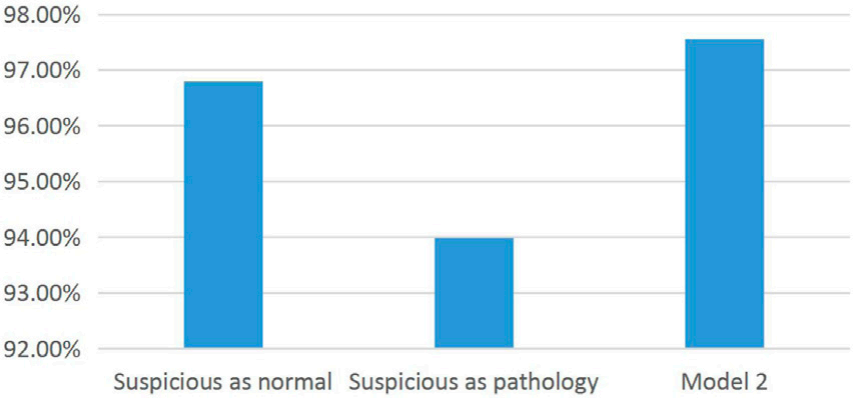
Histogram of the classification accuracy of the data set.

Figure 5 shows the results of dividing the CTG data set from three categories into two categories. The suspicious data were divided into health and pathological data through model 2, which improved the classification accuracy. At the same time, it also provides a discriminant method for a large number of suspicious data and reduces the second examination of patients. Suspicious data are regarded as pathology, and the classification accuracy is obviously low. Therefore, it also indicates that a large number of health data are contained in suspicious data. If not divided, a large number of healthy groups will receive unnecessary treatment and harm their health.

This experiment is comprehensively compared with other research methods, including the multi-layer perceptron neural network (MLPNN), probabilistic neural network (PNN), and generalized regression neural network (GRNN) proposed in the literature (y Formoso et al., 2020). The experimental results of different research methods on the data set are shown in (Table 8).

**TABLE 8:** Classification accuracy of different research methods on data sets.

	MLPNN (%)	PNN (%)	GRNN (%)	Ada-RF (%)
Classification accuracy	90.35	92.15	91.86	97.55

**FIGURE 6:**
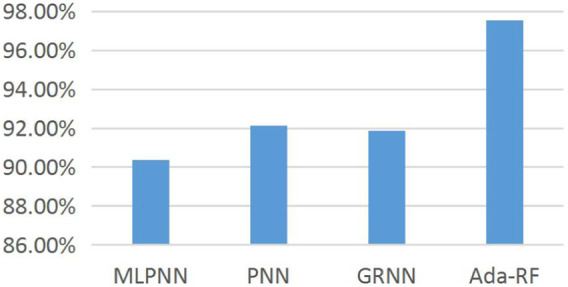
Histogram of the classification accuracy of different research methods on the data set.

It can be seen from Table 8 that the classification accuracy of the model in this study is higher than that of the other three models. Therefore, the classification accuracy of the overall data set is greatly improved by dividing the suspicious class data into health and pathology; at the same time, it also verifies the applicability of the feature selection method and classification prediction model proposed in this study. Figure 6 shows the histogram of the classification accuracy more directly demonstrates the effectiveness of the classification method in this study. The experimental results show that the combination of feature extraction based on the Apriori algorithm, and the classification prediction model has higher classification accuracy than other algorithms.

## 4 Conclusion

In this study, a feature selection, multi-model prediction, and classification method based on the Apriori algorithm are proposed to solve the intersection problem of suspicious data between health and pathological data. By dividing suspicious data into health and pathology, the classification accuracy of the whole dataset is greatly improved. At the same time, compared with other models, the proposed method has higher classification accuracy. The experimental results show that the feature extraction and model classification proposed in this study have good effects and are of great significance for clinical decision-making, healthy fetal development, and safe delivery of pregnant women. However, there are no real data to verify the prediction results for the prediction of suspicious data in this study, which is expected to be verified in future studies. In the future, feature extraction and classification will be carried out from the aspect of CTG signal processing to verify the classification of suspicious data sets so as to increase the authenticity of this study.

## Data Availability

The datasets presented in this study can be found in online repositories. The names of the repository/repositories and accession number(s) can be found in the article/Supplementary Material.
